# Combined dietary supplementation of long chain inulin and *Lactobacillus acidophilus* W37 supports oral vaccination efficacy against *Salmonella* Typhimurium in piglets

**DOI:** 10.1038/s41598-019-54353-1

**Published:** 2019-11-29

**Authors:** Alexia F. P. Lépine, Prokopis Konstanti, Klaudyna Borewicz, Jan-Willem Resink, Nicole J. de Wit, Paul de Vos, Hauke Smidt, Jurriaan J. Mes

**Affiliations:** 1 Immunoendocrinology, Division of Medical Biology, Department of Pathology and Medical Biology, University of Groningen, University Medical Center Groningen, Hanzeplein 1, 9700 RB Groningen, The Netherlands; 2 Food & Biobased Research, Wageningen University & Research, Bornse Weilanden 9, 6708 WG Wageningen, The Netherlands; 3 Laboratory of Microbiology, Wageningen University & Research, Stippeneng 4, 6708 WE Wageningen, The Netherlands; 4 Trouw Nutrition Research & Development, Stationsstraat 77, 3811 MH Amersfoort, The Netherlands

**Keywords:** Immunology, Microbiology, Zoology

## Abstract

Routine use of antibiotics in livestock animals strongly contributed to the creation of multidrug-resistant *Salmonella* Typhimurium strains (STM). Vaccination is an alternative to the use of antibiotics but often suffers from low efficacy. The present study investigated whether long-chain inulin (lcITF) and *Lactobacillus acidophilus* W37 (LaW37) can support vaccination efficacy against STM and if the interventions influence possible gut microbiota changes. Piglets received daily supplementation until sacrifice. Animals were vaccinated on day 25 after birth, one day after weaning, and were challenged with STM on days 52–54. Dietary intervention with lcITF/LaW37 enhanced vaccination efficacy by 2-fold during challenge and resulted in higher relative abundance of *Prevotellaceae* and lower relative abundance of *Lactobacillaceae* in faeces. Although strongest microbial effects were observed post STM challenge on day 55, transient effects of the lcITF/LaW37 intervention were also detected on day 10 after birth, and post-weaning on day 30 where increased relative abundance of faecal lactobacilli was correlated with higher faecal consistency. LcITF treatment increased post-weaning feed efficiency and faecal consistency but did not support vaccination efficacy. Vaccination in immune-immature young animals can be enhanced with functional additives which can simultaneously promote health in an ingredient-dependent fashion.

## Introduction

Antibiotics have been used in livestock for decades to prevent pathogenic infection and to promote animal growth^1^. This has contributed to the rise of antibiotic resistance^2^. Such global health issue has led to tighter regulations as illustrated by the 2006 EU ban on prophylactic use of antibiotics in livestock^3^. This ban, however, has led to increased therapeutic use of antibiotics and a subsequent rise in prevalence of resistant *Salmonella* in pigs^4^. Although pigs are mostly asymptomatic, *Salmonella* carriage, especially when multi-drug resistant, remains an important risk factor for meat contamination. Currently, non-typhoidal *Salmonella* is a major vector of multi-resistance genes as recently shown from isolates sampled in 20 hospitals of Thailand^5^, and is responsible for 9.3% of 225 foodborne outbreaks annually in Europe^6^. Therefore, there is an urgent need to develop alternative ways to prevent spread of *Salmonella* infections in livestock, for example by applying feed strategies to support immunity of the animals, or through use of vaccinations.

Vaccination of piglets against *Salmonella* occurs via the oral route but is not fully effective, conferring only ca. 50% protection^6,7^ and requiring several doses^8,9^. Conceivable approaches to increase vaccination efficacy might include simultaneous administration of dietary supplements known to enhance immunity^7,10–13^. Amongst the most extensively studied immune active agents are dietary fibres^12,14,15^ and lactic acid bacteria^16–18^, which have also been recognized as means to increase performance and well-being of piglets post-weaning and to reduce diarrhoea^19–21^.

Dietary fibres stimulate a stable and functional intestinal microbial community, and inulin-type fructans (ITF) are recognized prebiotic dietary fibres^22^ shown to support bifidobacterial growth and activity^23^. They are utilized and fermented by the intestinal microbiota leading to production of beneficial metabolites such as short-chain fatty acids (SCFA) and support the growth of beneficial *Bifidobacterium* communities^24^. As previously described by Vogt *et al*.^25^, ITF are also immunomodulatory in addition to their indirect prebiotic effects^25–27^. More specifically, long-chain ITF (lcITF) may support immunity against non-typhoidal *Salmonella enterica* subsp. *enterica* serovar Typhimurium (STM) as it triggers a type 1 helper T cell (Th1) skewing during vaccination^25,28^. Therefore, lcITF might support other Th1 based vaccination protocols such as those targeting STM^29^.

Amongst lactic acid bacteria, direct introduction of live *L. acidophilus* has been associated with enhanced health status^30^, reduced shedding of pathogens^31^ and disease symptoms^32,33^, and support of intestinal immunity^34,35^. *L. acidophilus* was also shown to induce Th1 cytokines in mice^36^, to increase IFN-γ producing T cells, and to reduce Treg in gnotobiotic pigs^37^. The probiotic strain *L. acidophilus* W37 (LaW37) is therefore another ingredient that might be supportive in preventing STM infection. This specific strain LaW37 supported barrier integrity of epithelial cells during STM challenge *in vitro*^38^. Furthermore, complementary effects were observed on TLR activation *in vitro* with LaW37 and lcITF in a dose-dependent fashion, and depending on the receptor^39^. For instance, TLR3 was activated by LaW37 alone but not by lcITF alone, whereas TLR5 was strongly activated by lcITF and not by LaW37. As there was no counter effect on these activations when combining the two treatments at their highest concentrations, this combination is likely to have additive effects.

Dietary interventions and vaccination are applied orally, potentially interacting with the gastrointestinal microbiota, and thus affecting the immune and metabolic status of the animals in later life^40–43^. Although the effect of *Salmonella* vaccination^6^ and dietary interventions^43–45^ on swine microbiota development have been studied already, the effect of combining both has, to the best of our knowledge, never been studied. Intestinal colonization starts at birth to reach adult-like intestinal microbiota by 3–4 weeks post-weaning in pigs^46–48^. The adult pig microbiota typically comprises genera *Clostridium*, *Blautia*, *Lactobacillus*, *Prevotella*, *Ruminococcus*, *Roseburia*, the RC9 gut group and *Subdoligranulum*^46,49–51^. Members of the families *Enterobacteriaceae* and *Bacteroidaceae* have been reported to be amongst the most abundant at birth; however, their relative abundance decreases with weaning, whereas that of *Prevotellaceae* increases, becoming the most abundant family after weaning^46,51^. During the vulnerable neonatal phase, piglets are protected by maternal antibodies while their intestinal immune system develops and matures^52–54^. Host-microbiota interactions in the developing gut are considered critical during this stage, as any perturbation can potentially lead to impaired immune function later in life^40,55^. Vaccination is commonly performed pre-weaning, but possible interference with microbiota colonization, and the effect of dietary interventions, is unknown.

The hypothesis was that lcITF and LaW37 combined might uniquely support oral vaccination efficacy against STM, as well as reduce severity of the infection. Effects of lcITF supplementation, alone or combined with LaW37, were investigated on STM oral vaccination efficacy in piglets and on intestinal microbiota development. A suboptimal dose of the vaccine was given to facilitate read out of beneficial effects of dietary supplementation on STM vaccination.

## Results

### Well-being of piglets was specifically affected by vaccination and dietary supplementations

LcITF alone or combined with LaW37 was studied for its effect on vaccination efficacy in piglets. Composition of the different groups is presented in Table 1. General well-being of the animals was analysed as secondary outcome.

**Table 1:** Treatment groups. lcITF = long-chain inulin type fructans; LaW37 = *L. acidophilus* W37.

Group	Supplementation	Vaccination	Challenge	Number of animals
Negative control	Placebo	No	Yes	7
Positive control	Placebo	Yes	Yes	6
lcITF	0.114 g/d/kgBW	Yes	Yes	8
lcITF/LaW37	0.114 g/d/kgBW + 5 × 10^9^ CFU/d/piglet	Yes	Yes	7

Before weaning, lcITF improved general health status (*p* = 0.029, Table 2), which was scored daily looking at signs of dehydration, meagreness, skin colour and activity levels. This was not observed in the lcITF/LaW37 group. Pre-weaning mortality was recorded and reached 27.5% in the CTRL group and 20% in both supplemented groups.

**Table 2:** General health parameters followed from birth until challenge.

Groups	1	2	3	4	P-level treatments
Treatments (number of animals)	CTRL/NV (n = 7)	CTRL/V (n = 6)	lcITF/V (n = 8)	lcITF/LaW37/V (n = 7)	
**Farrowing room (prior to weaning and to vaccination) d0–23**					
Growth performance (g/day)	256 ± 8		238 ± 9	242 ± 7	0.189
Health condition* (% of all scores > 0)	7.7 ± 5^**b**^		4.1 ± 3^**a**^	6.7 ± 4^**ab**^	**0.029**
**First week post-weaning d24–30**					
Growth (g/day)	49 ± 18	52 ± 20	86 ± 15	42 ± 11	0.215
Feed intake (g/day)	101 ± 15	93 ± 15	112 ± 12	86 ± 11	0.482
Feed efficiency (g feed/g weight)	0.60 ± 0.06^**b**^	0.61 ± 0.08^**b**^	0.82 ± 0.06^**a**^	0.54 ± 0.06^**b**^	**0.034**
Faecal score** (% of piglets with soft stools to diarrhoea)	23.6%	20.5%	10.3%	13.8%	0.1718
**Post weaning period until challenge d24–51**					
Health condition* (% of animals scoring > 0, with 0 the ideal state)	17.3 ± 3^**a**^	31 ± 3^**b**^	34.9 ± 2^**b**^	29.4 ± 4^**b**^	**<0.0001**
Appetite*** (% of animals scoring > 0, with 0 the ideal state)	20 ± 3^**a**^	27 ± 3^**b**^	31 ± 3^**b**^	26 ± 4^**b**^	**0.004**
Faecal score** (% of piglets with soft stools to diarrhoea)	22.9%^**a**^	19.7%^**a**^	12.2%^**b**^	20.8%^**a**^	**0.0115**

Different letters indicate significant difference between the groups (*p* < 0.05) when the general *p* value is below 0.05 as indicated in bold. Statistical significances were tested with a χ^2^ homogeneity test of the GENMOD procedure in SAS. CTRL = placebo control; NV = non-vaccinated; V = vaccinated; lcITF = long-chain inulin type fructans; LaW37 = *L. acidophilus* W37.

^*^Score 0 = good condition; 1 = below average; 2 = less optimal; 3 = meagre; 4 = dehydrated.

**Incidence of diarrhoea evaluated with faecal score 1 = soft; 2 = diarrhoea; 3 = severe dehydration.

***Score 0 = normal appetite; 1 = little appetite; 2 = no appetite.

At weaning, vaccination decreased the general health score (*p* < 0.0001) and lowered the appetite (*p* = 0.004, Table 2). These effects could neither be attenuated by lcITF nor lcITF/LaW37. However, in this post weaning phase, lcITF, but not lcITF/LaW37, increased feeding efficiency (*p* = 0.034) compared to CTRL/NV (Table 2).

Overall, incidence of faecal scores 1–3 was low post-weaning from d24–51, until start of the challenge, but significantly lower in animals receiving lcITF compared to all other groups (*p* = 0.011, Table 2). Faecal shedding of live STM during challenge was followed as an indicator of piglets’ infectious state. Shedding remained low and was not influenced by lcITF or lcITF/LaW37 (data not shown). Translocation of STM in piglets’ spleen and tonsils was also low (data not shown) confirming the mild impact of the STM challenge on the animals. No mortality was recorded after weaning, nor during challenge.

### Microbiota was transiently and specifically affected by the combination of dietary interventions with vaccination but not by vaccination itself

**Figure 1:**
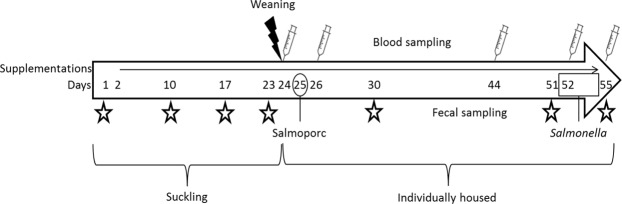
Experimental design. Female piglets were cross-fostered d1. Supplementation via oral drenches of placebo, inulin (lcITF) or lcITF/*L. acidophilus* (LaW37) started on d2 and were continued daily until sacrifice, on d55 after birth. Piglets testing *Salmonella*-free were weaned on d24 and vaccinated on d25 with Salmoporc. Oral challenge with *Salmonella* Typhimurium (STM) of animals confirmed to be *Salmonella*-free prior to challenge was given daily on d52, d53 and d54. The animals were sacrificed on d55. Blood samples (syringes) were collected on d24, d26, d44 (20 days post vaccination), prior (d52) and post (d55) STM challenge. Faecal samples (stars) were collected shortly after birth, on d10, d17, d23, d30, d51 and d55.

Faecal sampling was also performed to determine the main factors influencing microbiota development from birth to d55, through weaning, potentially including vaccination, in the presence and absence of dietary supplementation. The experimental design is further detailed on Fig. 1. Diet together with age was the main driver, as distinct differences were observed between microbiota composition of samples prior- and post-weaning (Supplementary Fig. S1A; Bray-Curtis). Overall, microbiota was more diverse post-weaning, reflecting the transition to solid diets (Supplementary Fig. S1B., Shannon index). This was also observed using other measures of alpha diversity, including phylogenetic diversity, InvSimpson and Observed OTU’s indices (data not shown).

**Figure 2:**
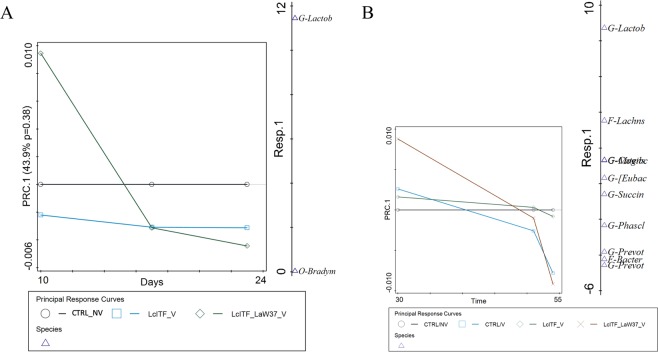
PRC analysis for the effect of dietary intervention during the (**A**) pre- and (**B**) post-weaning periods, reveal microbial changes one week after weaning (d30) and upon STM challenge (d55). (**A**) PRC analysis during pre-weaning was performed on microbiota data collected on d10, d17 and d21. (**B**) PRC analysis during post-weaning was performed on microbiota data collected on d30, d51 and d55. CTRL = placebo control; NV = non-vaccinated; V = vaccinated; lcITF = long-chain inulin type fructans; LaW37 = *L. acidophilus* W37.

Furthermore, the influence of the dietary interventions on early life microbiota was evaluated prior to, during and after weaning, and in relation to administered oral vaccination. In order to address more specifically the evolution of microbial composition under the influence of the different dietary interventions throughout time, principal response curve analysis (PRC) was performed (Fig. 2). The model did not identify a significant effect of any of the dietary treatments, at any of the pre-weaning time-points, which was also observed with alpha-diversity based on Shannon index (Supplementary Fig. S2A–C). However, on d10 PRC revealed a higher relative abundance of lactobacilli in lcITF/LaW37 compared to control and lcITF groups, although not significant (Fig. 2A).

**Figure 3:**
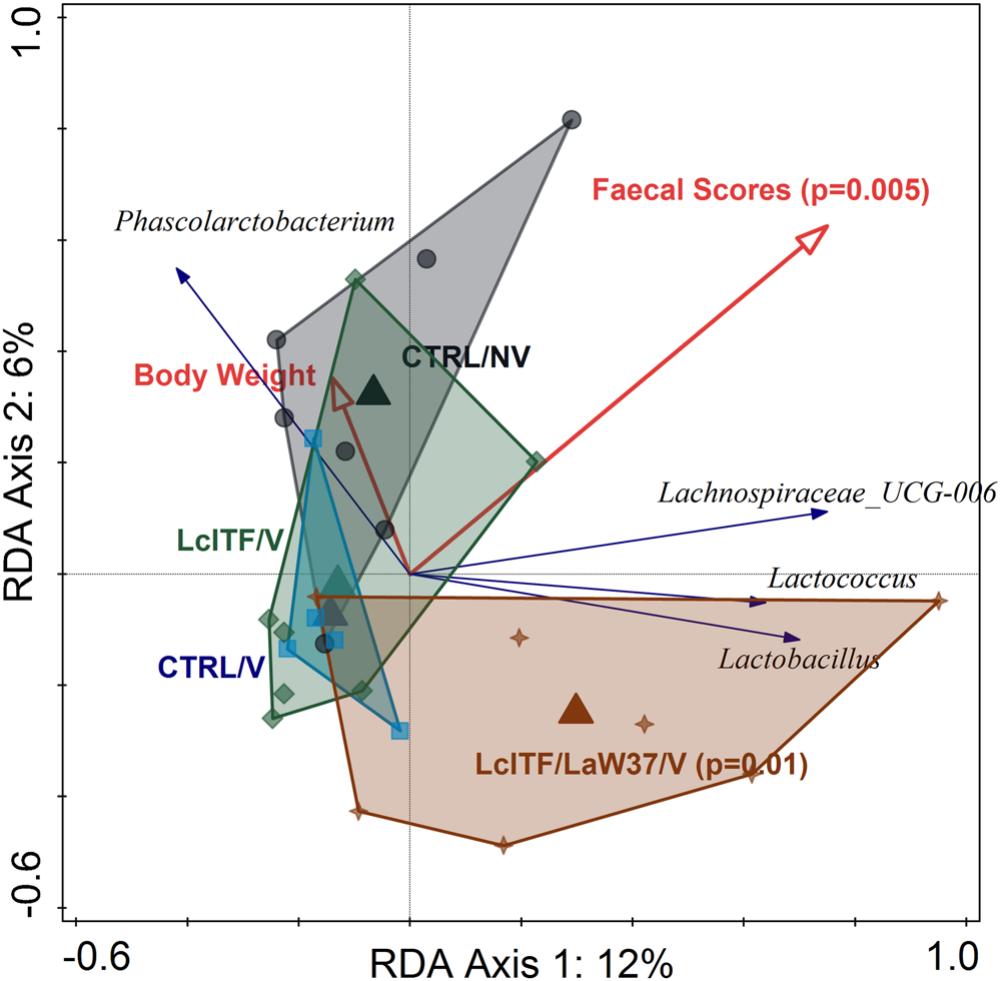
RDA triplot showing the associations between the faecal microbiota composition and environmental variables on day 30. Triangles represent different experimental groups, red arrows numerical environmental variables and blue arrows the 4 best fitting bacterial genera on d30. The plotted first and second ordination axes explain 12% and 6% of the variability in the dataset. Samples are coloured by treatment CTRL = placebo control; NV = non-vaccinated; V = vaccinated; lcITF = long-chain inulin type fructans; LaW37 = *L. acidophilus* W37.

PRC analysis of post-weaning samples showed that microbiota composition was significantly affected by the different dietary treatments, accounting for 37.4% of the variation (*p* = 0.01). Prior to STM challenge differences were observed on d30 but not d51 (Fig. 2B), and no differences were observed in alpha-diversity on either of these days (Supplementary Fig. S2D,E). The significant deviation between the different groups on d30 was further supported using unconstrained ordination based on Bray-Curtis dissimilarity (R^2^ = 0.20, *p* = 0.007; ADONIS). To more specifically test the effect of experimental variables, constrained redundancy analysis (RDA) was applied where the lcITF/LaW37/V group separated from the other three groups (*p* = 0.002), explaining 7.6% of the variation (Fig. 3). The sum of faecal scores collected from weaning (d24) until d30 were found to explain 10.8% of the variation in microbial composition observed on d30 (*p* = 0.005, Fig. 3). Importantly, soft stools and mild diarrhoea recorded from d24–30 were higher in control groups compared to lcITF/V and lcITF/LaW37/V groups, albeit not significantly (Table 2). Animals receiving lcITF/LaW37/V were characterized by higher faecal relative abundances of lactobacilli (Supplementary Fig. S3A), which were also positively correlated with faecal scores (*p* = 0.06) within the lcITF/LaW37/V group (Supplementary Fig. S3B). This effect of lcITF/LaW37/V on d30 was transient and no separation could be observed on d51 (data not shown).

In conclusion, vaccination itself, as observed when comparing the control groups, did not impact microbiota development, and neither did the combination of vaccination with lcITF alone. In contrast, vaccination combined with the dietary intervention lcITF/LaW37 affected microbial composition on d30.

### Only LcITF/LaW37/V enhanced efficacy of STM vaccination

Animals were vaccinated after confirmation of *Salmonella*-free status, with a single dose (1/3 of the oral vaccination protocol) Salmoporc STM on d25, and antibody titres were analysed just before and after vaccination, and during the following weeks. All animals were orally challenged with STM on d52. The challenge was applied daily for three consecutive days and blood was sampled for antibody titres prior to and post challenge.

**Figure 4:**
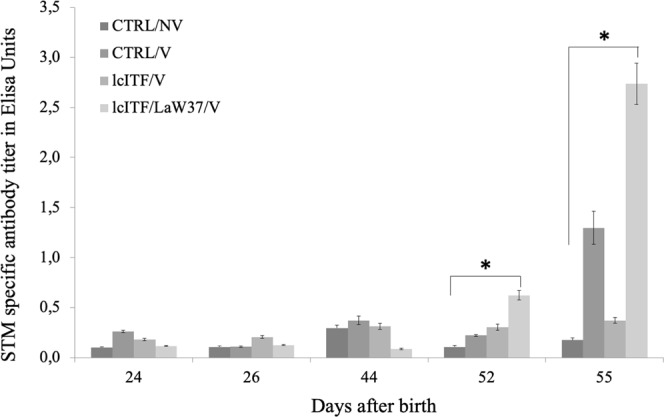
Levels of antibody titres in piglets’ blood after vaccination against *Salmonella* Typhimurium in the different treatment groups. Antibody titer was measured prior to vaccination on d24, a day after vaccination (d26) and on d44, d52 and d55 after birth. Challenge with STM started on d52, after blood samples were collected, and ended on d54. Animals were sacrificed on d55, after blood samples were collected. Significant differences with CTRL/NV tested with Kruskal-Wallis, followed by a Dunn’s test in GraphPad Prism (*p* < 0.05), are indicated by *. Data are expressed as mean ± SEM. CTRL = placebo control; NV = non-vaccinated; V = vaccinated; lcITF = long-chain inulin type fructans; LaW37 = *L. acidophilus* W37.

Only the lcITF/LaW37 treatment resulted in significantly higher antibody titres compared to CTRL/NV both on d52 (*p* = 0.020), and on d55 (*p* = 0.003) (Fig. 4). Interestingly, antibody titres in CTRL/V animals was not significantly higher than in the CTRL/NV group indicating that a suboptimal vaccination protocol was indeed used.

Besides effects on vaccination efficacy, possible underlying mechanisms were tested by studying immune related parameters in blood. Most striking effects on immune cells were observed during STM challenge, therefore only these data are shown in Table 3.

**Table 3:** Effects of the dietary interventions on immune parameters during STM challenge period. Frequency of immune cells in piglets’ blood was measured using flow cytometry. CTRL = placebo control; NV = non-vaccinated; V = vaccinated; lcITF = long-chain inulin type fructans; LaW37 = *L. acidophilus* W37.

Marker	Time point	CTRL/NV	CTRL/V	LcITF/V	LcITF/LaW37/V
Monocytes/granulocytes	No effect observed				
NK CD56dim	No effect observed				
NK CD56bright	Difference between d52 and d55	0.0311*	0.0515*	0.0347*	0.0086
CTLs CD3+ CD8+	d55	28.4^ab^	33.65^a^	21.30^b^	28.15^a^
Th CD4+ CD8+	d55	24.81^a^	26.37^a^	35.84^b^	30.16^a^
CD3+ CD8+ CD45RO+	d55	2.57	1.68	2.84	4.14^#^
CD4+ CD8+ CD45RO+	d55	4.78	3.89	4.97^#^	5.16^#^

*Statistically significant difference (p < 0.05) between d52 and d55 in frequency of NK CD56 bright cells within a treatment group.

Different letters (a,b) represent statistical significance (p < 0.05).

^#^Indicates a trend (p < 0.1) between the treatment group and the CTRL/V group.

Levels of NK CD56 bright cells, involved in pathogen responses, were similar for all groups until STM challenge, where they decreased significantly for CTRL/NV, CTRL/V and lcITF/V (*p* = 0.0071) but not in animals supplemented with lcITF/LaW37/V (*p* = 0.568). Also on d55, frequency of cytotoxic T lymphocytes (CD3^+^ CD8^+^) (CTLs) was lower in the lcITF/V group than in the CTRL/V group (*p* = 0.011). This decrease was not found with lcITF/LaW37/V (*p* = 0.115). Similarly, Th cell (CD4^+^ CD8^+^) frequency was higher in lcITF compared to CTRL/NV (*p* = 0.004), CTRL/V (*p* = 0.028), and lcITF/LaW37/V (*p* = 0.014).

CD45RO^+^ memory T cells found in the CTLs and Th populations were subsequently measured. LcITF/LaW37/V tended to have twice as many memory CTLs than CTRL/V (*p* = 0.111). In the Th compartment, the effect of the supplements was similar for lcITF/V and lcITF/LaW37/V, where frequency of CD45RO^+^ cells was higher for both groups compared to CTRL/V (*p* = 0.073).

Together, these data point at ingredient-dependent effects on piglets’ immune parameters after a challenge, indicating a ‘primed’ or ‘trained’ type of immune modification.

### Microbiota was differently affected by STM infection depending on the dietary intervention

**Figure 5:**
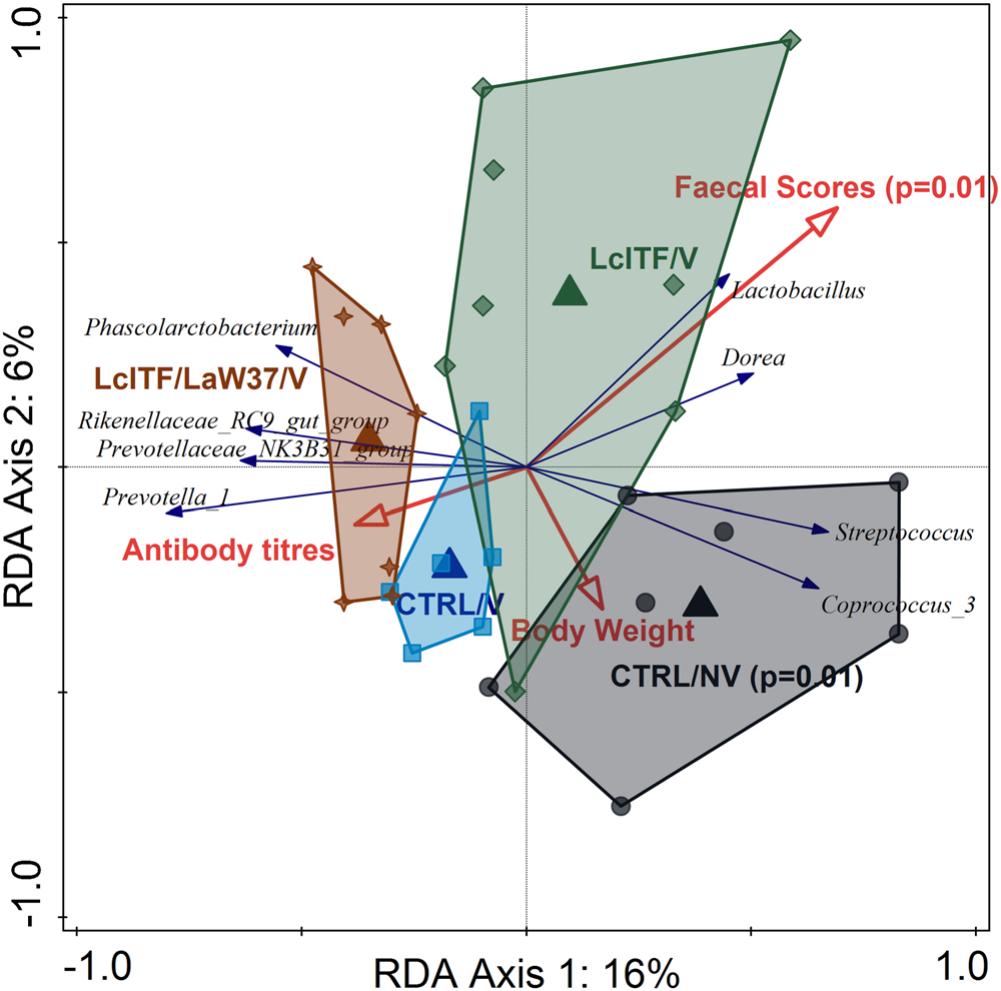
Effect of STM challenge on faecal microbiota composition was different depending on the treatments. (**A**) RDA triplot showing the association between microbiota and environmental variables using samples from day 55, post STM challenge. Triangles represent different experimental groups, red arrows numerical environmental variables and blue arrows the 8 best fitting bacteria. The plotted first and second axes explain 16% and 6% of the variation in the dataset. Samples are coloured by treatment CTRL = placebo control; NV = non-vaccinated; V = vaccinated; lcITF = long-chain inulin type fructans; LaW37 = *L. acidophilus* W37.

A strong effect of the different dietary treatments on faecal microbiota composition could be observed on d55 (Fig. 2B). Unconstrained Bray-Curtis analysis showed that differences between the four groups were significant (R^2^ = 0.41; *p* = 0.001; ADONIS). Moreover, the groups lcITF/LaW37/V and CTRL/V shared high similarities in comparison with CTRL/NV and lcITF/V, on d55 as observed with constrained RDA analysis (Fig. 5). The CTRL/NV group had a distinct microbiota composition from the other groups, which were all vaccinated (10.4%, *p* = 0.002).

Moreover, increased faecal scores significantly explained part of this variation (9.2%, *p* = 0.002) and were mainly associated with lcITF/V (Fig. 5). Relative abundance of *Dorea* (R = 0.74; *p* = 0.019) and *Lactobacillus* (R = 0.62; *p* = 0.044) correlated with faecal scores in the lcITF/V group (Fig. 5; Supplementary Fig. S4A,B). The CTRL/NV group had significantly higher relative abundances of the genus *Streptococcus* in comparison with the other three groups and of the genus *Coprococcus* compared to CTRL/V and lcITF/LaW37/V groups but not lcITF/V (Fig. 5; Supplementary Fig. S4C,D). Moreover, the CTRL/NV group had the lowest alpha diversity (Supplementary Fig. S2F). Finally, antibody titres were associated with the microbiota of animals receiving lcITF/LaW37/V, although not significantly (Fig. 5).

The CTRL/V and LcITF/LaW37/V groups, of which microbiota composition separated from CTRL/NV and lcITF/V in the RDA analysis, presented higher relative abundances of members of the *Prevotellaceae* family, i.e. *Prevotella* 1, *Prevotellaceae*_NK3B31_group, but also from the genera *Phascolarctobacterium* and *Rikenellaceae*_RC9_gut_group (Fig. 5; Supplementary Fig. S5A–D). Moreover, microbiota changes prior to and post challenge, were more pronounced in the CTRL/NV and lcITF/V groups than in CTRL/V and lcITF/LaW37/V, however, these changes did not correlate significantly with antibody titres (Supplementary Fig. S6).

This suggests that during challenge the changes in piglets’ microbiota were more pronounced in non-vaccinated animals and vaccinated animals that received lcITF than in vaccinated control animals and those receiving lcITF/LaW37.

## Discussion

In this study it was shown that a combination of a dietary fibre (lcITF) and a lactic acid bacterium (LaW37) can enhance antibody titres after oral vaccination of piglets. Animals receiving lcITF had enhanced survival rates, improved general health before weaning and better feeding efficiency during weaning stress although there were only eight animals in this group. Antibody titres against *Salmonella* (STM) were doubled by lcITF/LaW37, and microbiota composition of the seven animals receiving that treatment, post STM challenge, was more preserved as compared to pre-challenge profiles. These data therefore confirm the hypothesis that this combination supports immunity and might be an instrumental alternative to the use of antibiotics in piglets^56,57^.

The lack of supportive effect of lcITF supplementation on vaccination efficacy was not expected. Earlier findings showed that lcITF supports Hepatitis B vaccination by increasing Th1 cells^28^, and STM vaccination is also known to be Th1 driven. In contrast, short-chain ITF was previously found to have suppressive effects against Hepatitis B vaccination^28^. It should be noted, however, that that study targeted a systemic vaccination in adults^28^ whereas the present design with oral vaccination targeted juveniles. Metabolism of long chain compounds such as lcITF in juvenile and mature individuals might differ, based on the differences observed between the results presented here and the ones of Vogt *et al*.^28^. However, it is important to note that dietary lcITF intervention in piglets was not associated, until challenge, with impaired general health. Moreover, analysis of T cells showed that lcITF alone, as response to STM challenge, decreased the number of CTLs and increased the number of T helpers. Although the specific population of Th cells presently increased could not be identified, these observations indicate that lcITF might have anti-inflammatory properties in piglets, stimulating a more Th2 response^45^, which was observed for short chain ITF^28^ and in previous trials with various inulins in pigs^14,45,58^.

Vaccination efficacy was doubled by the combination lcITF/LaW37 upon secondary exposure and was already increased prior to challenge, on d52, in this group. Faster build-up of immunity is considered to be advantageous for weaning piglets. While studies on ITF during vaccination protocols are scarce, many vaccination trials have investigated *Lactobacillus* effects. Most studies used systemic vaccination protocols with variable success^59,60^. To a lesser extent, probiotics have been tested in mucosal vaccination protocols, and all have obtained promising results^61–64^ in line with the present study. Interestingly, previous *in vitro* work on the combination lcITF/LaW37 has shown that these ingredients can have complementary effects^39^. Moreover, the strategy applied in the present study of a suboptimal vaccination protocol was meant to create a sufficient window to study effects of the dietary interventions, which would not be possible if the vaccination efficacy was directly triggering maximum production of antibody titres. All these factors are likely to contribute to the remarkable increase of antibody titre observed in animals receiving lcITF/LaW37. Oral vaccination based on live STM is a mild type of infection, and the immune system of piglets is likely to undergo a faster development when exposed to infections^65^. This might explain the difference observed in some immune parameters upon challenge. For instance, decreased levels of cytokine producing NK CD56-bright cells did not occur in lcITF/LaW37 treated piglets. A possible explanation, in line with the fact that piglets treated with lcITF/LaW37 responded most to the vaccination, is that the development of their immune system was influenced by the concomitant exposure to the vaccine and LaW37 which could possibly lead to differentiation of NK CD56bright towards other NK types^66^.

Shedding and translocation in spleen or tonsils were low, indicating that the challenge was mild. However, it is during challenge that most effects of the dietary interventions were observed on microbiota. In this period, clear distinctions between CTRL/V and lcITF/LaW37/V on one side, and CTRL/NV on the other side, could be observed with *Prevotellaceae* and *Lactobacillaceae* as main drivers for these differences. On day 55, four days after the STM challenge, alpha diversity in the CTRL/NV group decreased, as previously observed^67,68^. Moreover, relative abundances of *Streptococcus* in the CTRL/NV group were significantly higher compared to CTRL/V and lcITF/LaW37/V groups indicating that this typical colonizer of the pigs’ upper intestinal tract^69,70^ were being excreted. Interestingly, relative abundance of other upper tract colonizers from the family *Lactobacillaceae* was higher in the CTRL/NV and lcITF/V groups, and both CTRL/NV and lcITF/V experienced a significant shift in microbiota composition during the STM challenge. *Salmonella* is known to invade ileal mucosa provoking diarrhoea^71^ therefore inducing loss of microbial diversity. Ileal microbiota composition can then be measured in faecal samples within the first days of an STM infection^67,68^, which would typically be rich in lactobacilli^69,70^. Interestingly, the present analysis revealed that faecal scores were positively associated with the lcITF/V group, and positive correlations between *Dorea* and *Lactobacillus* with faecal scores were found in the CTRL/NV and lcITF/V groups. Therefore, the present data indicate that lcITF/V animals had intestinal dysbiosis upon STM challenge and were more similar to CTRL/NV than to the other vaccinated groups, in terms of faecal microbiota composition, in line with antibody titre levels.

Animals that received CTRL/V and lcITF/LaW37/V were more protected against STM-induced dysbiosis as their microbiota composition was preserved upon STM challenge, but microbiota was not solely responsible for this. These two groups clustered together based on their microbiota composition and were typically characterized by higher relative abundance of *Prevotellaceae* and lower *Lactobacillaceae. Prevotella* typically colonizes the cecum and colon of healthy pigs^69^, and higher levels have been associated with health^72^. Changes in *Prevotellaceae* were previously observed in pigs infected with *Salmonella*^67,68^. This is further supported by the present observations as animals with higher *Prevotellaceae* also experienced stronger systemic reaction towards the STM-challenge as measured by increased antibody titre. However, this association was not significant, and a significant increase of antibody titre was only observed in animals receiving lcITF/LaW37/V and not CTRL/V. The present data therefore suggest that changes in faecal microbiota cannot solely explain the doubled antibody titre measured in lcITF/LaW37/V as compared to the CTRL/V group, which was similarly protected against STM-induced dysbiosis observed in CTRL/NV. Interestingly, in a recent study, *Prevotella* was found to be less abundant in *Salmonella* infected pigs compared to noninfected pigs during the weaning and growing stages^73^. The authors of this study suggested that a lack of microbiota maturation increased susceptibility to infection and that modifying certain taxa within the porcine intestinal microbial community could result in and increased disease resistance against *Salmonella*. The findings of this and the present study should be addressed in more mechanistic studies, including e.g. experiments where piglets are inoculated with defined microbial communities of increasing complexity and degree of maturation, in order to elucidate potential causal relationships between the kinetics of early life colonization with specific microorganisms and microbial consortia and susceptibility for *Salmonella* infection.

In conclusion, chronic dietary intervention in piglets with lcITF and lcITF/LaW37 is not only safe, it can also be efficacious and might help to reduce the need for therapeutic antibiotic treatments thereby limiting associated undesirable effects. Despite the immaturity of the immune system of piglets, the combination lcITF/LaW37 enhanced oral STM vaccination efficacy. Also, weaning and STM challenge but not the vaccination itself affected faecal microbiota composition. The present data reinforces the importance of carefully selecting dietary supplements for enforcing specific desired immune and microbiota responses^74^. LcITF was beneficial at weaning but not to support vaccination efficacy while LaW37 had no additional effect besides a strong enhancement of vaccination efficacy, whereby the mechanisms behind this effect do not solely rely on changes in faecal microbiota. The data presented here illustrates that effects of food ingredients on immunity are very specific and cannot be effective without a rational design^27,28,74^.

## Methods

### Ethical statement

The experiment was designed in compliance with guidelines for animal research, and experiments were performed under DEC committee approval no. DEC 2012.III.05.041. The description of application can be found on page 301 under ‘Veevoederbedrijf’ of the yearly report (http://dierproefinfo.nl/dec/decabc-2012.pdf).

### Supplements, vaccine and challenge compounds

LcITF (Frutafit TEX! Sensus, Roosendaal, the Netherlands) isolated from chicory roots contained oligomers and polymers with degree of polymerization from 10 to 60, linked by β(2–1) bonds. LcITF was characterized by high-performance anion exchange chromatography coupled with pulsed electrochemical detection (HPAEC-PED), which was performed on an ICS5000 system (Thermo Fisher Scientific, Waltham, MA, USA), equipped with a Dionex CarboPac PA-1 column (2 × 250 mm) in combination with a Carbopac PA-1 guard column (2 × 50 mm) (Supplementary Fig. S7).

LaW37 (Winclove, Amsterdam, The Netherlands) was produced anaerobically at 37 °C in media adapted from Man Rogosa Sharpe broth.

STM strain DT12 (B; O1, 4, 5, 12) was isolated from a pig mesenteric lymph node^75^. Inocula were prepared as previously described^76^ and were used to challenge the piglets. In short, bacteria were grown from glycerol stocks in Brain-Heart Infusion medium at 37 °C until stationary phase. Cell count was confirmed with plating on Columbia Blood Agar medium.

Salmoporc STM is an oral live attenuated porcine vaccine licensed in Europe (IDT Biologica, Dessau-Roßlau, Germany). The lot number used was 0161213, and vaccine suspension was prepared freshly, according to manufacturer’s instructions, prior to administration.

### Experimental procedures

Twenty-eight Hypor*Maxter newborn female piglets were selected from eighteen sows housed at Trouw Nutrition Research & Development (Sint Anthonis, The Netherlands) and randomly allocated to one of the following four treatments: 1. control non-vaccinated (CTRL/NV) included eight piglets, 2. control vaccinated (CTRL/V) included six piglets, 3. inulin vaccinated (lcITF/V) included eight piglets and 4. lcITF combined with *L. acidophilus* W37 vaccinated (lcITF/LaW37/V) included seven piglets. To avoid as much as possible confounding effects such as genetic background, maternal antibodies and differences in maternal microbiota, four piglets from each of the selected sows were cross-fostered after 24 hours, on day 1 after birth (d1), before assignment to the treatment groups (Table 1). Minimization of sow effects was confirmed (Supplementary Table S1). Cross-fostering occurred within 12–14 piglets standardized litters, and each sow fostered an equal number of piglets that received the same treatment to avoid cross-contamination. Researchers and farm technicians were blinded for treatment, and the experiment was conducted in a single period with the indicated number of animals.

Suckling piglets were kept together with their fostering mother, each sow being housed in farrowing pens with steady temperature, humidity and light. No creep feed was supplied to the piglets. From weaning on, piglets were individually housed at the health care unit. Animals accessed *ad libitum* water and feed, which was a synthetic diet low in fibre adapted from Houdijk *et al*.^77^ (Supplementary Tables S2 and S3) and produced by Trouw Nutrition.

Supplementations of lcITF or lcITF/LaW37 were administered daily to the selected piglets by oral gavage, starting on d2. LcITF in sterile PBS was administered at 0.114 g/kg BW. Lyophilized LaW37 was used in a fixed dose of 5 × 10^9^ CFU/piglet. It was added to the lcITF within 1 h prior to gavage. Glucidex 2 (Roquette Corporate, Lestrem, France) and starch carrier served as placebo for the control groups.

Piglets were weaned on d24, after they were confirmed to be free of culturable *Salmonella* spp. In their faeces. On d25 animals received one dose of Salmoporc STM oral vaccination of approximately 10^9^CFU/piglet. The specific responses against *Salmonella* were tested by oral administration of 10^9^CFU of STM DT12 per piglet (GD Animal Health, Deventer, The Netherlands) suspended in 1 mL PBS for three constitutive days, d52, d53 and d54. Absence of non-vaccine *Salmonella* was confirmed prior to challenge.

Blood was collected five times from the jugular vein on d23, d25, d42, d52, d55 (Fig. 1), at a set time early in the morning prior to any other handling of the animals, for antibody titre and flow cytometric analysis. Blood was collected at these time points with sterile S-Monovette lithium-heparinized tubes (Sarstedt AG & Co, Numbrecht, Germany).

Faecal samples were collected on d23, prior to weaning and vaccination, on d52, prior to challenge, and at 24 h, 30 h, 48 h and 72 h of STM challenge for *Salmonella* CFU count. Besides, faeces were collected for microbiota analysis via rectal stimulation shortly after birth and at set times in the morning on d10, d17, d23, d30, d51 and d55 (Fig. 1). Faecal samples were stored in sterile tubes at −20 °C until further processing.

Zootechnical parameters included the following factors (Supplementary Text S1). Health status and faecal scores of the animals were evaluated daily. Appetite was rated only in weaner piglets and feed intake was calculated by weighing the feed left, on d30, d33, d38, d45, and daily during the *Salmonella* challenge (d52, d53, d54). The body weight was measured at birth, 24 h after birth, on d10, d17, d23, d30, d33, d38, and d45, prior to (d51) and post (d55) challenge with STM. Feed efficiency was calculated in weaner piglets as ratio of feed intake and weight gain.

The study ended on d55 when animals were euthanized with an overdose of barbiturate by intra-cardiac injection following a stratified randomization sequence. From each animal, tonsils, ileum, and feces were taken for CFU count of STM.

### Serology

Blood was centrifuged at 2,000 *g* for 10 min and plasma was stored at −80 °C until further use. Detection of anti-*Salmonella* antibodies was performed with Salmotype Pigscreen ELISA according to manufacturer’s instructions (Labordiagnostic Leipzig, Leipzig, Germany). Specific IgG levels were calculated using a reference standard method and are presented as S/P values.

### Flow cytometry

Granulocytes and monocytes (CD172b+), NK cell (CD56+), T lymphocytes (CD3+), cytotoxic T cells (CD3+CD8+) and T helper cells (CD3+CD4+) were stained in whole blood. Expression of CD45RO (memory T cells) was measured within CD8+ and CD4+ subsets. Specification of the antibodies used is shown in Supplementary Table S4 and gating strategy is shown on Supplementary Fig. S8. See Supplementary Text S2 for the procedure.

### Salmonella occurrence in piglet faeces and tissues

Absence or presence of STM in piglets’ faeces was determined on d24, prior to vaccination, and on d52, prior to challenge. All animals were salmonella-free prior to STM challenge on d52. Furthermore, during the challenge, live STM was quantified in faeces collected at 24 h, 30 h 48 h, and 72 h after the beginning of the challenge and at sacrifice (d55). *Salmonella* colonies were counted as previously described^78^ and expressed as CFU/gram. Of each sample, two presumptive *Salmonella* colonies were confirmed by qPCR for both *Salmonella* and STM. When no colonies were observed on the lowest dilution plates, the samples were screened for *Salmonella* presence/absence after pre-enrichment by the conventional Modified Semi-Solid Rappaport Medium/Xylose Lysine Deoxycholate method. One colony on the Xylose Lysine Deoxycholate plate was again confirmed by qPCR.

Typing was performed on random colonies isolated from faeces, preceding the challenge, to discriminate the vaccine strain from possible contamination. This was performed by the Netherlands National Institute for Public Health and the Environment (RIVM, The Netherlands) following an optimized multiple-locus variable number tandem-repeat assay for characterization of STM as previously described^79^.

### Data normalization and statistics

Antibody titres were not normally distributed as confirmed by D’Agostino & Pearson normality test and were further analysed using Kruskal-Wallis followed by Dunn’s multiple comparison test in GraphPad Prism version 7.0a (GraphPad Software). As results of Dunn’s test, *p*-values of 0.05 or smaller were considered statistically significant and *p*-values between 0.05 and 0.1 were defined as a trend. Data are expressed as mean ± standard error of the mean (SEM).

Flow cytometry data was analysed with one-way ANOVA followed by LSD *post-hoc*, in GraphPad Prism. Data within a time-point was defined as independent while data recorded for a specific animal throughout time was analysed as paired. *P*-values of 0.05 or smaller were considered statistically significant and *p*-values between 0.05 and 0.1 were defined as a trend. Data are expressed as mean ± SEM.

The zoological, clinical parameters and *Salmonella* quantification in faeces, tonsils and ileum samples were tested in a Proc MIXED procedure in SAS 9.3 Software Version 13. (SAS Institute Inc., Stata Corporation, College Station, TX, USA) according to SAS/STAT 9.3 User’s Guide using the following equation:*Y*_*ijk*_ = *μ* + *T*_*i*_ + *e*_*ijk*_ where T is the treatment effect for each group (1,2,3,4), and fostering sow is taken as a random factor. Feces consistency, and health scores were analyzed with a χ2 homogeneity test of the GENMOD procedure in SAS. In this case, weekly frequency of each score was used for the Genmod procedure as frequencies read to model the probabilities of score levels having lower ordered values in the response.

### HiSeq sequencing of the 16S rRNA gene V4 region

Bacterial DNA was isolated from approximately 0.1 g of faecal material that was diluted in 350 μL of STAR buffer (Roche Diagnostics GmbH, Mannheim, Germany) and homogenized (Bertin Technologies, CNIM, Montigny-le-Bretonneux, France) (3 × 5.5 m/s for 30 s) with 0.25 g of sterilized 0.1 mm diameter zirconia/silica beads (Sigma) and 3 glass beads (2.5 mm). Homogenized samples were incubated at 95 °C for 15 min and centrifuged for 5 min (4 °C/13,000 *g*). Supernatants were pooled and DNA was purified using the Maxwell R16 Instrument (Promega, Leiden, The Netherlands) as described previously^80^. Purified DNA was quantified using a DeNovix DS-11 (DeNovix Inc., Wilmington, USA) spectrophotometer, and aliquots of 20 ng/ μL for each sample were prepared using nuclease free water and used for later PCR amplification steps as described in Supplementary Text S3.

### Microbiota composition analysis

Sequence data filtering and taxonomy assignment were performed using the NG-Tax pipeline^81^. Sequences were filtered to contain only read pairs with perfectly matching barcodes and were assigned to Operational Taxonomic Units (OTU) excluding low abundant OTUs (less than 0.1%) from each sample. Taxonomy was assigned using the Silva128 reference dataset^82^. In total 218 samples were sequenced in four batches and a total of 42,748,032 sequence reads were obtained. To assess the batch effect, we examined the distribution of the reads per library which was highly similar between the four libraries suggesting lack of batch effect in terms of number of reads between the four libraries (Kruskal Wallis, *p *= 0.38) (Supplementary Fig. S9A–C). Moreover 24 randomly selected samples were sequenced as duplicates (Supplementary Fig. S10) and results showed high similarity (Pearson: R = 0.92–0.99; Supplementary Table S5). Finally, 17 samples were sequenced as positive controls, using two distinct in-house assembled mock communities^81^. The mock communities presented identical composition regardless of the batch they were included in (Pearson: R = 0.96–0.99; Supplementary Fig. S11A,B), and presented strong correlations with their theoretical composition (Pearson: R = 0.76 for Mock 3 and Pearson: R = 0.84 for Mock 4). Alpha and beta diversity analyses were performed using microbiome R package version 1.1.2^83^. To determine significance for the effect of the environmental variables within each timepoint, Adonis Permutational Analysis of variance was conducted using the dissimilarities from the Bray-Curtis index. Principal response curve (PRC) and redundancy analyses (RDA) were performed using CANOCO 5, to test for treatment effects^84^. RDA is a multivariate analysis where multiple response parameters can be related to a set of environmental variables. PRC is used for analysis of treatment effects in experiments with a repeated measures design. The analyses in CANOCO were conducted using the genus level relative abundances. False discovery rate (FDR) was used to determine the significance of explanatory variables.

## Data availability

The 16S rRNA gene sequences are publicly available in the European Nucleotide Archive (ENA) under code PRJEB31925 https://www.ebi.ac.uk/ena/data/view/PRJEB31925. The authors declare that all other data supporting the findings of this study are available within the paper and its additional files or from the corresponding authors upon reasonable request.

## Supportung Information

Supportung Information
