# Supplementary material for: Combined dietary supplementation of long chain inulin and Lactobacillus acidophilus W37 supports oral vaccination efficacy against Salmonella Typhimurium in piglets
Source: Sci Rep. 2019 Nov 29;9:18017. doi: 10.1038/s41598-019-54353-1 (PMC6884548; doi:10.1038/s41598-019-54353-1)
Supplement: Supplementary file 1 — Supportung Information [file 41598_2019_54353_MOESM1_ESM.pdf]

Combined dietary supplementation of long chain inulin and *Lactobacillus acidophilus* W37 supports oral vaccination efficacy against *Salmonella* Typhimurium in piglets

Alexia F. P. Lépine[1,2,†], Prokopis Konstanti[3,†], Klaudyna Borewicz[3], Jan-Willem Resink[4], Nicole J. de Wit[2], Paul de Vos[1,#], Hauke Smidt[3,#], and Jurriaan J. Mes[2,*,#]

[1] Immunoendocrinology, Division of Medical Biology, Department of Pathology and Medical Biology, University of Groningen, University Medical Center Groningen, Hanzeplein 1, 9700 RB Groningen, The Netherlands
[2] Food & Biobased Research, Wageningen University & Research, Bornse Weilanden 9, 6708 WG Wageningen, The Netherlands
[3] Laboratory of Microbiology, Wageningen University & Research, Stippeneng 4, 6708 WE Wageningen, The Netherlands
[4] Trouw Nutrition Research & Development, Stationsstraat 77, 3811 MH Amersfoort, The Netherlands
† These authors equally contributed
# These authors equally contributed

Supplementary Text S1

Health status of the animals was evaluated daily by the same blinded and trained farm technician throughout the whole study. Health status included monitoring of behaviour, body condition and skin condition. Animals drop-out was also reported. Behaviour was rated on a scale from 0 to 4, where 0 is a communicative, alert and playful piglet, 4 is a pathetic and passive behaviour, and intermediate scales are 1 a calm animal, 2 a slow animal, and 3 a listless animal. To evaluate that, the researcher observed the interaction between the piglets within a litter and curiosity towards himself and other litters. Body condition was rated on a scale from 0 to 4 where 0 is a healthy animal with excellent physical condition, a good fleshiness rounded shape of the back and clear development of the ham. A sick animal would be rated 4 being really thin with visible backbone without flesh and showing signs of dehydration. Intermediate scores were 1 for intermediate animals without much ham development, 2 for thin animals without much flesh on the back and the ribs and 3 meagre animals with visible backbone. This scale was based on a portfolio so that all the conditions described above were illustrated by example pictures. Scores displayed in the tables are % of the incidence of scores 1+2+3+4.

Faecal consistency was scored daily in the morning (DMS-00446 SRC Faeces protocol Trouw Nutrition, The Netherlands). It was scored per litter prior to weaning and per animal post weaning. The scale ranges from 0 to 3, 0 being the ideal well shaped stool with soft but solid consistency and 3 being a severe diarrhoea with light coloured watery defecation. Intermediate scales go from 1 with soft sticky stool to liquid consistency. Results are presented as percentage of weekly incidence within one treatment group.

Appetite was rated only in weaner piglets based on belly fill and eagerness when the feed was given to them. Feed intake was calculated by weighing the feed left, on days 30, 33, 38, 45, and daily during the *Salmonella* challenge on days 52, 53, 54 and 55. The body weight was measured (DMS-00293 SRC Weighing procedure, Trouw Nutrition, The Netherlands) at birth, 24 hours after birth, on days 10, 17, 23, 30, 33, 38, and 45, prior to challenge with STM, and at the moment of sacrifice. Feed efficiency was then calculated in weaner piglets as ratio of feed intake and weight gain.

Feed intake was calculated by weighing the feed left, on days 30, 33, 38, 45 after birth, and daily during the *Salmonella* challenge on days 52, 53 and 54 after birth. The body weight was measured at birth, 24 hours after birth, on days 10, 17, 23, 30, 33, 38, and 45 after birth, prior to challenge with STM, and at sacrifice. Feed efficiency was calculated in weaner piglets as ratio of feed intake and weight gain.

Supplementary Text S2

After a 1:1 dilution in RPMI (Lonza, Basel, Switzerland) supplemented with 10 % heat inactivated Foetal Calf Serum (hiFCS) (HyClone, GE Healthcare Life Science, Utah, USA), the cells present in 0.5 mL blood were pelleted and incubated for 30 min in 100 µL in FACS buffer (PBS + 10 % hiFCS (v/v)) supplemented with 10 % (v/v) mouse serum (Sanquin, Jackson lab, Amsterdam, The Netherlands) and containing the antibody mix (Supplementary Table S4). Next, the cells were incubated with a biotinylated antibody (streptavidin-Brilliant Violet 785) for 15 min to label the CD56 antibodies. The erythrocytes were then lysed with 2 mL FACS Cell Lysing Solution (BD Bioscience, Breda, The Netherlands) and the leukocytes were fixed with 300 µl CellFIX (BD). Washing was performed between all incubation steps and every incubation step was carried out at 4°C in the dark.

Stained cells were analysed using the LSR-II Flow Cytometer System (BD Bioscience, Breda, The Netherlands), using FACS Diva software. Analysis was performed using FlowJo version 10 software (FlowJo, LLC, Oregon, USA). Approximately $5x10^6$ cells were recorded, and frequency of each population was expressed as % of the parent population. The gating strategy for monocytes, NK cells and T lymphocytes are displayed in Supportive Figure S8.

All singlets were selected based on size in forward side scatters (FSC) of area (A) and width (W). Lymphocytes were selected using FSC/CD172a plots as CD172a negative cells. NK cells were selected from the lymphocyte plot as $CD3^-$ cells and $CD56^+$ cells. The T cells are $CD3^+$ cells, within which $CD4^+$ and $CD8^+$ were selected. Expression of $CD45RO^+$ was measured within both these populations using the zebra plots so that the lower limit of the gates was set on the upper line of the centre core.

Supplementary Text S3

The PCR reactions were carried out in duplicate using 20 ng of DNA as template in each 50 µL reaction. One µL of each of the primers 515-n and 806-n, targeting the V4 region of 16S ribosomal RNA (rRNA) gene region and uniquely barcoded per sample (10 µM each), was used along with, 1 x HF buffer (Finnzymes, Vantaa, Finland), 1 µL dNTP Mix (10 mM each, Roche), 1 U Phusion® Hot Start II High Fidelity DNA Polymerase (Finnzymes, Vantaa, Finland) and 36.5 µL of DNAse and RNAse free water. The amplification program included 30 s initial denaturation step at 98°C, followed by 25 cycles of denaturation at 98°C for 10 s, annealing at 50°C for 10 s, elongation at 72°C for 10 s, and a final extension step at 72°C for 7 min. PCR products were visualized by agarose gel electrophoresis, and PCR product size (~290 bp) was compared to a 1 kb DNA ladder (ThermoScientific, Waltham, MA, USA). PCR products from each sample duplicate were pooled, purified using magnetic beads CleanPCR kit (CleanNA, Alphen aan den Rijn, The Netherlands) and eluted in 30 µL of nuclease-free water (Qiagen). The DNA concentration of each sample was determined with Qubit BR dsDNA assay kit (Thermo Fisher Scientific, Waltham, MA, USA) and 100 ng of purified DNA were used for the construction of amplicon libraries. In total 218 samples were sequenced, distributed in four libraries of 70 samples, labelled with uniquely barcoded primers. Final amplicon pools were concentrated using magnetic beads and eluted in 25 µL of nuclease free water and the libraries were sent for Illumina HiSeq sequencing (GATC- Biotech, Konstanz, Germany).

Supplementary Figures

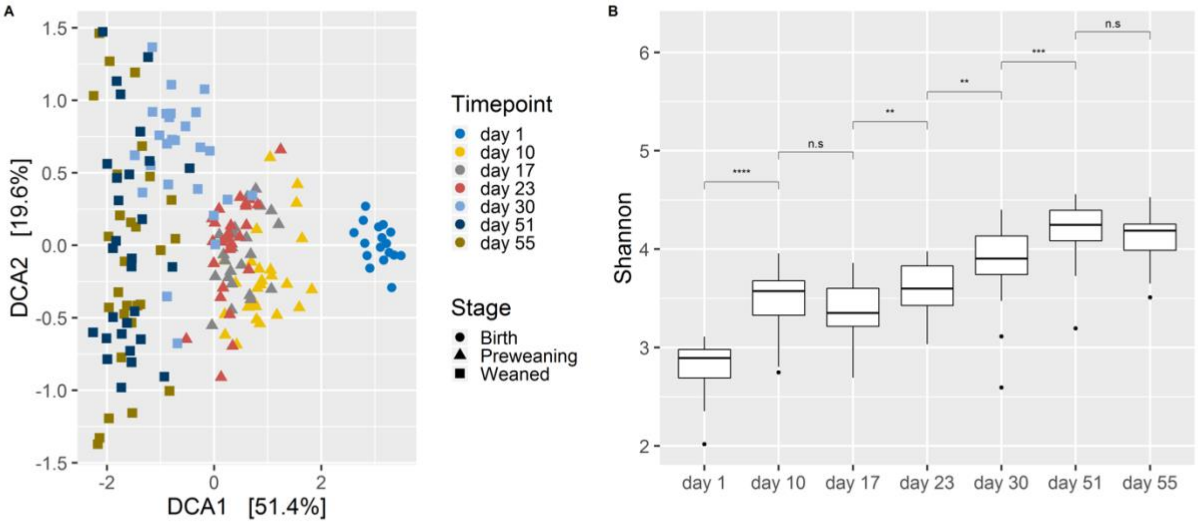

Figure S1. The development of microbial communities in neonatal piglets from birth to day 55 after birth. A) Detrended Correspondance Analysis plot based on pairwise Bray-Curtis distances, illustrating the succession of microbial development in piglets. Percentages of variation are indicated for each axis. B) Shannon diversity of the developing microbial communities of piglets.

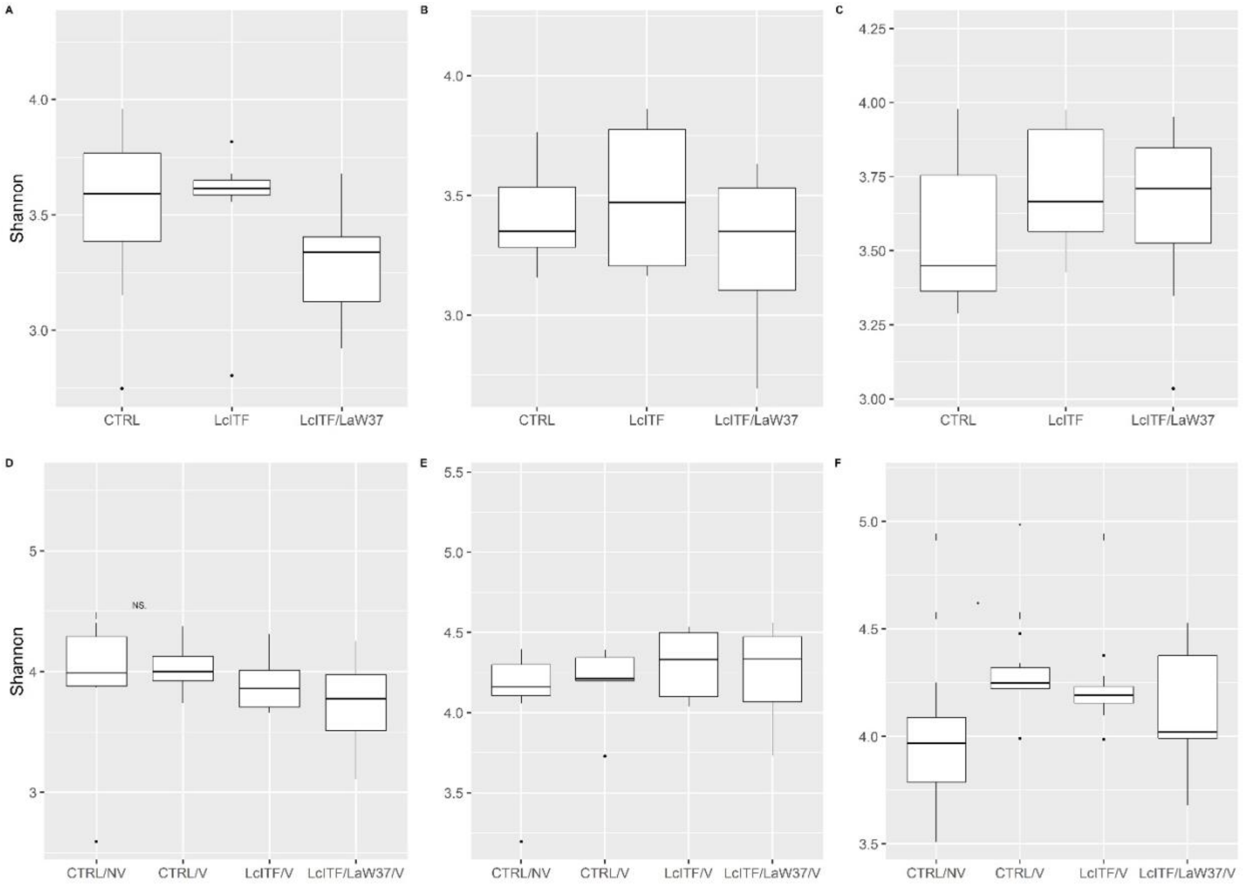

Figure S2. Alpha diversity measurements per group. Alpha diversity was estimated within each timepoint using the Shannon diversity index. (A-C) Results for the faecal samples collected in the pre-weaning period A) on day 10, B) day 17 and C) day 24. (D-F) Results for the faecal samples collected in the post-weaning period D) on day 30, E) day 51 and F) day 55. Abbreviations stand for placebo = control; NV = non-vaccinated; V = vaccinated; lcITF = long-chain inulin type fructans; LaW37 = *Lactobacillus acidophilus* W37.

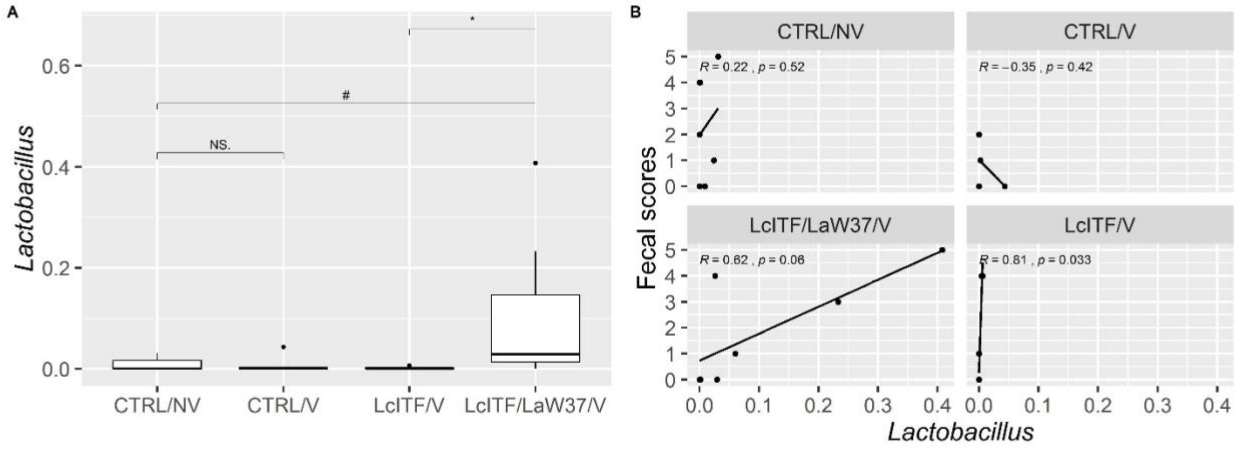

Figure S3. A) Box plot with relative abundances of the genus *Lactobacillus* on d30 between the four groups tested, where # $p<0.1$ and * $p<0.05$. B) Significant correlations with faecal scores are shown with cross correlation between scores and the genus Lactobacillus for each group tested using Kendall's tau coefficient. Abbreviations stand for placebo = control; NV = non-vaccinated; V = vaccinated; lcITF = long-chain inulin type fructans; LaW37 = *Lactobacillus acidophilus* W37.

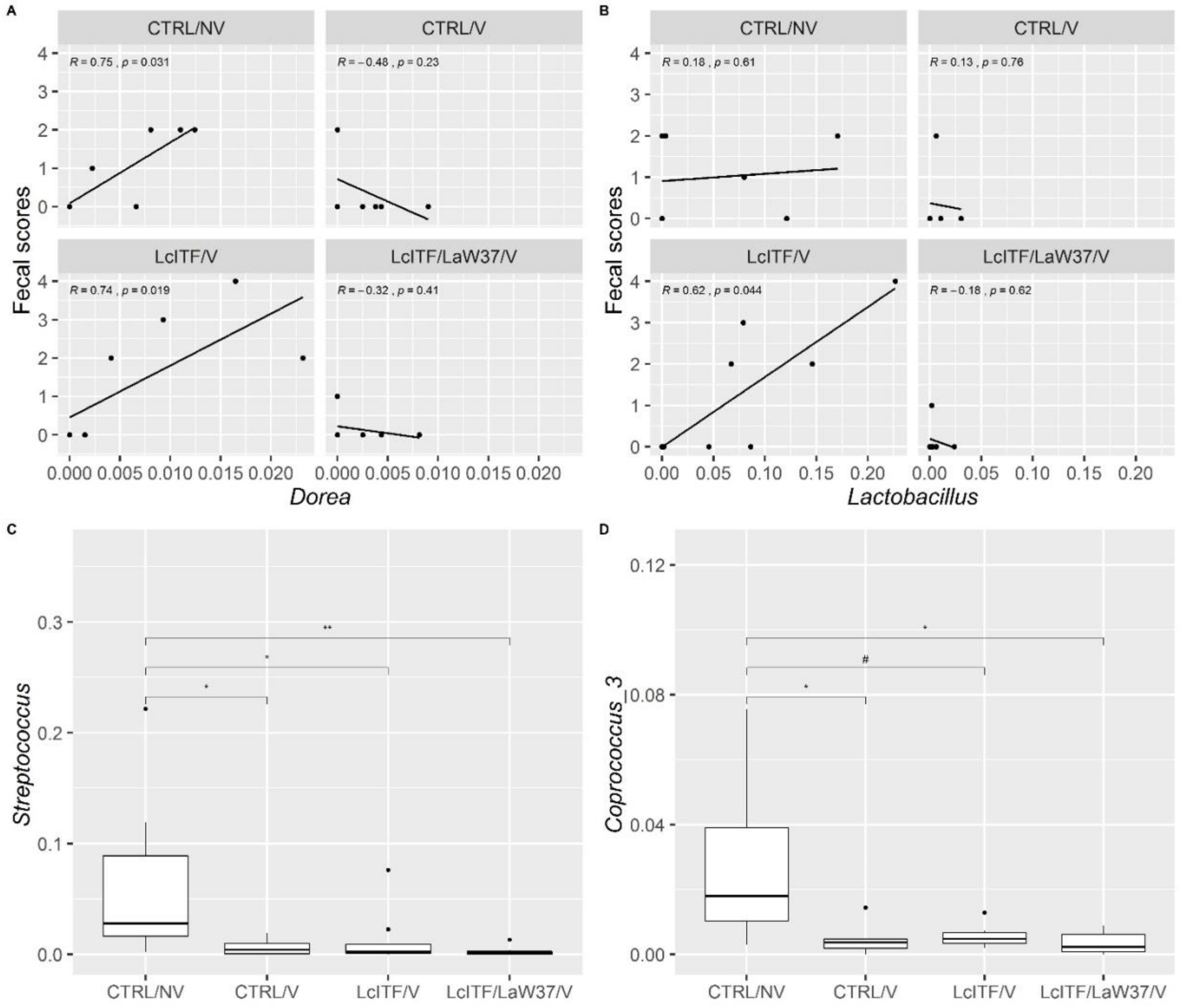

Figure S4. Kendall cross correlations between faecal scores and the genera A) *Dorea* and B) *Lactobacillus* for each group tested on day 55. Tau coefficients are indicated for each group along with the p-value. C-D Box-plots showing the interquartile range (IQR) of the relative abundances of the genera *Streptococcus* and *Coprococcus* both of which were increased in the non-vaccinated group on day 55. CTRL=placebo control; NV=non-vaccinated; V=vaccinated; lcITF=long-chain inulin type fructans; LaW37=*L. acidophilus* W37.

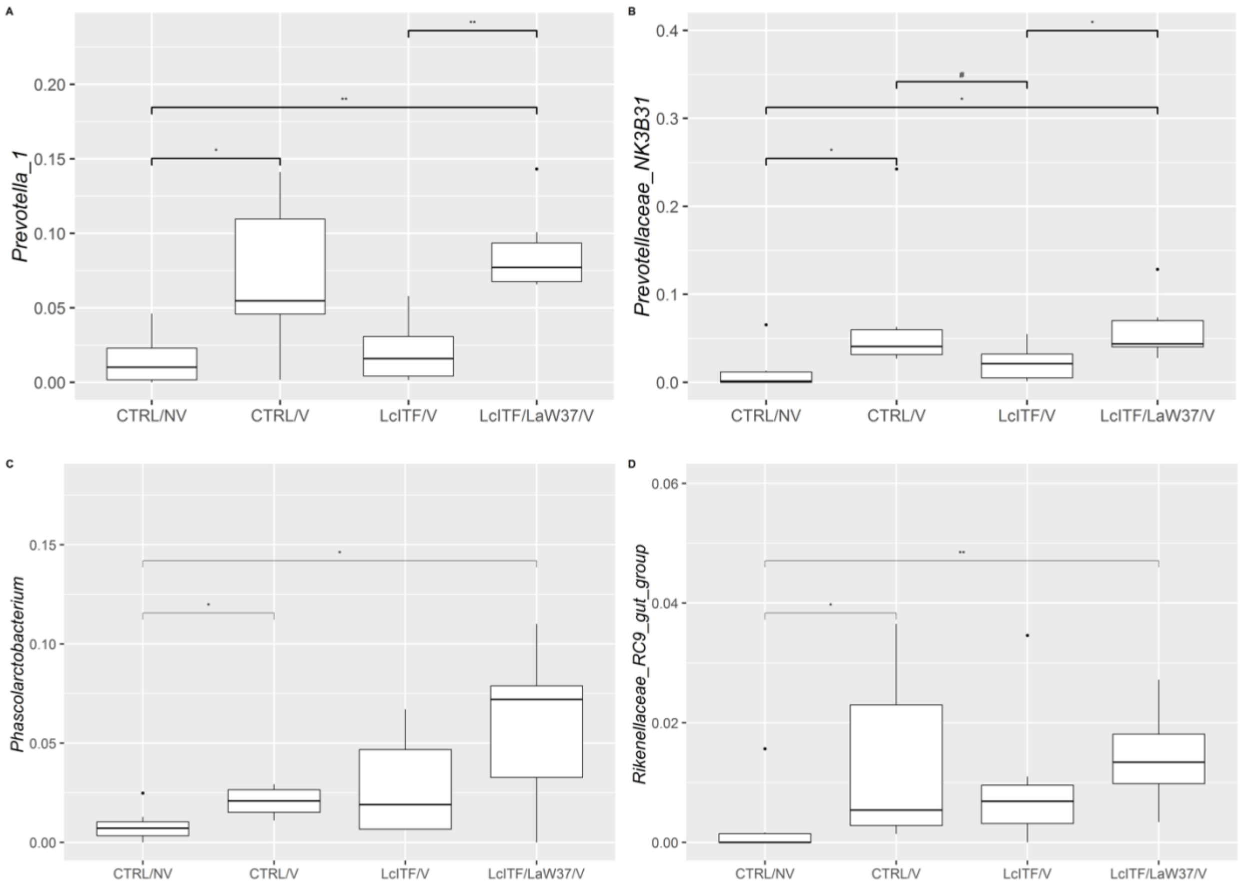

Figure S5. The relative abundances of the bacterial genera which were enriched in the lcITF/LaW37/V and CTRL/V groups on day 55 upon *Salmonella* Typhimurium challenge. Box-plots showing the interquartile range (IQR) of the relative abundances of the genera A) *Prevotella_1*, B) *Prevotellaceae_NK3B31_group*, C) *Phascolarctobacterium* and D) *Rikenellaceae_RC9_gut_*group. Abbreviations stand for CTRL = placebo control; NV = non-vaccinated; V = vaccinated; lcITF = long-chain inulin type fructans; LaW37 = *Lactobacillus acidophilus* W37.

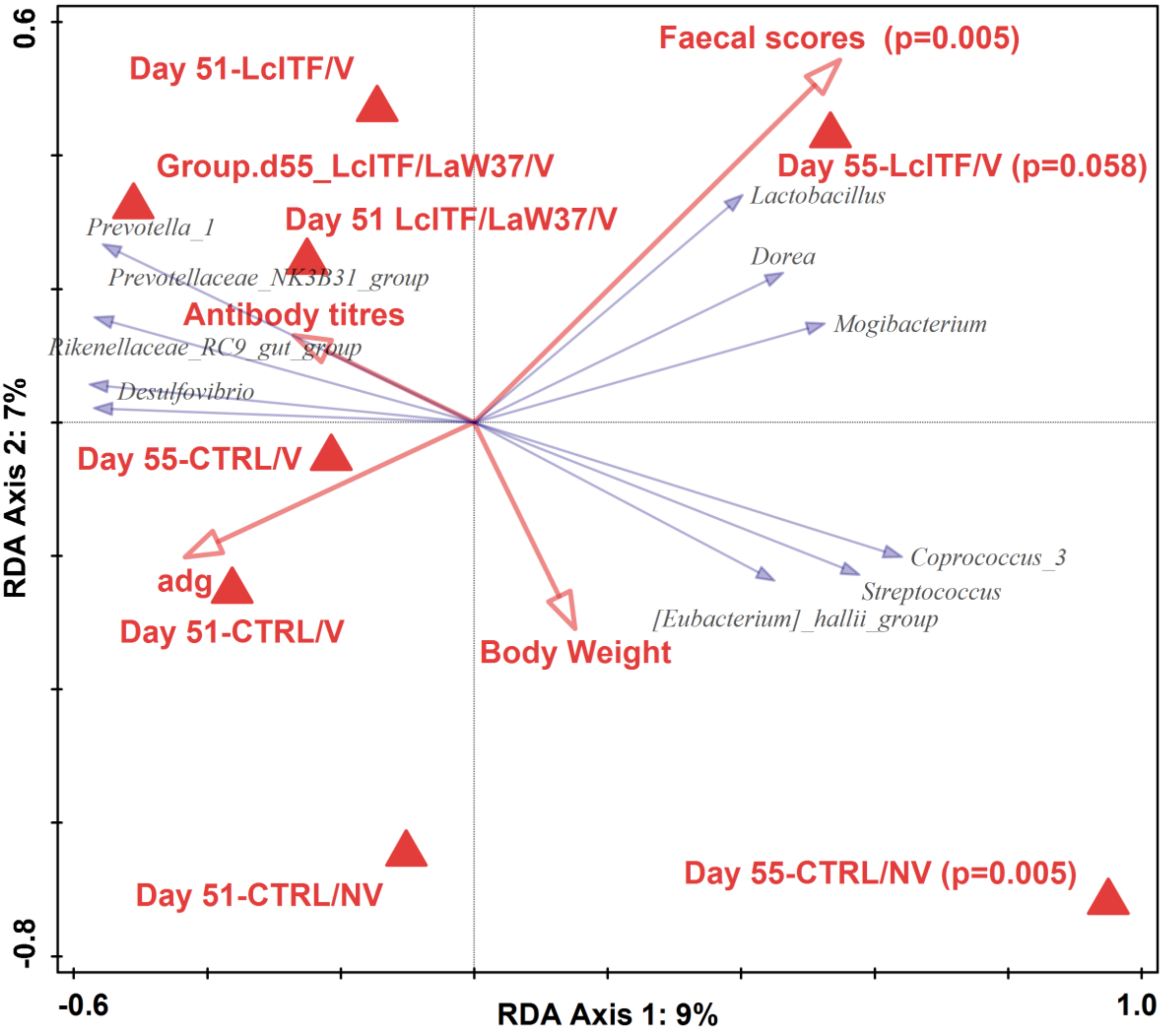

Figure S6. RDA biplot showing the association between the faecal microbiota composition and the environmental variables using samples prior (d51) and post (d55) STM challenge. Triangles represent different experimental groups, red arrows numerical environmental variables and blue arrows the 10 best fitting bacteria. The plotted first and second axes explain 9 % and 6 % of the variation in the dataset. Associations are shown with the 10 best fitting bacterial genera. Abbreviations stand for CTRL = placebo control; NV = non-vaccinated; V = vaccinated; lcITF = long-chain inulin type fructans; LaW37 = *Lactobacillus acidophilus* W37.

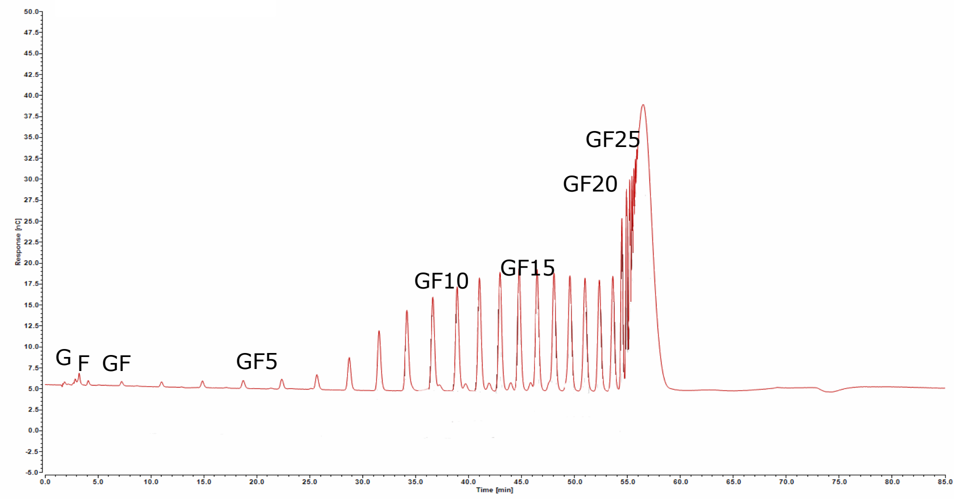

Figure S7. Long-chain inulin-type fructans (lcITF; Frutafit® TEX!) HPAEC profile. Peaks represent fructose (F) and glucose (G) monomers, dimers and fructan oligomers present in the formulation of lcITF. GFn and Fn chains respectively terminated by a glucose or fructose molecule with n the number of fructose moieties in the chain.

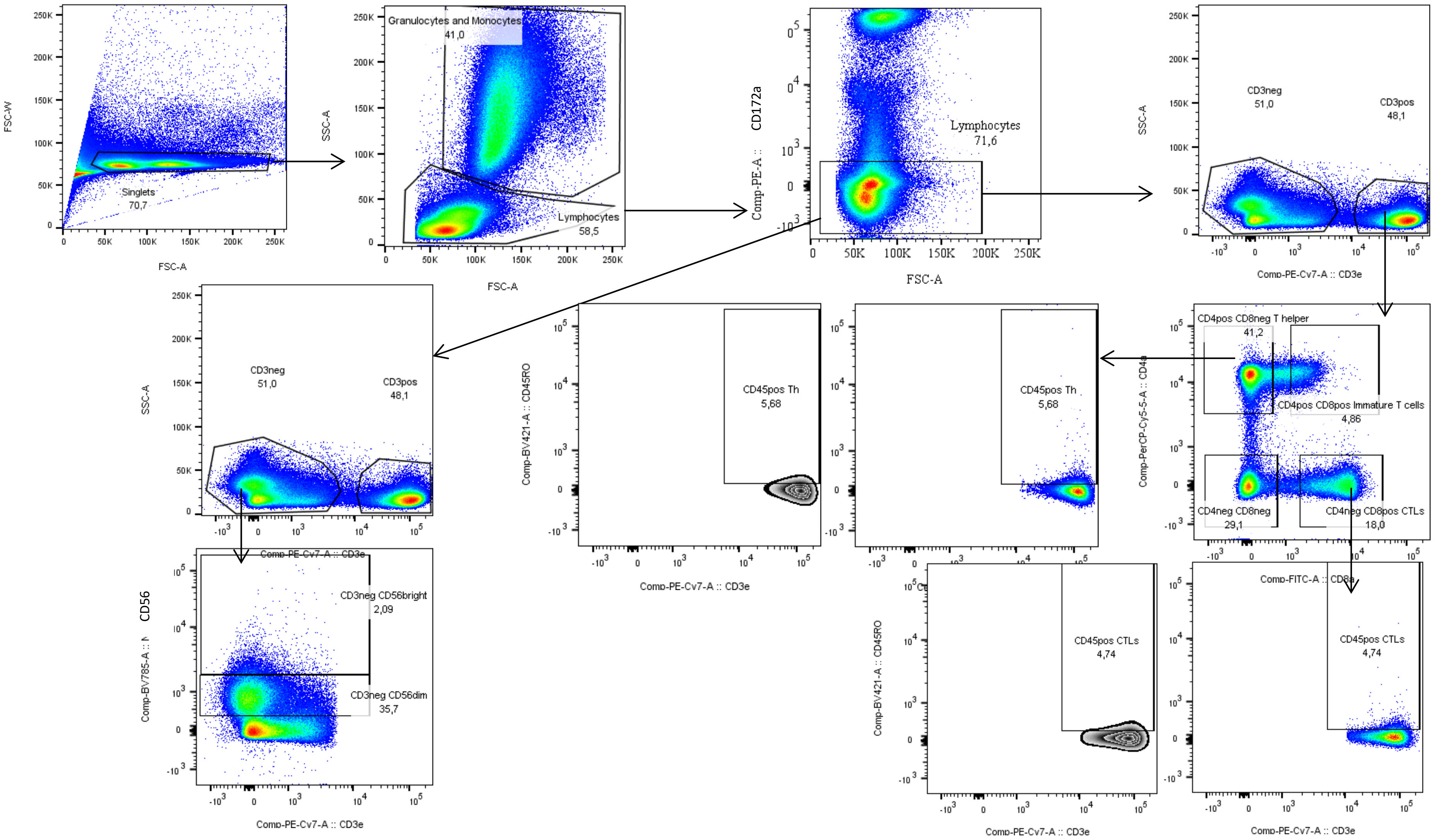

Figure S8. Gating strategy for determination of NK cells and T cells subsets in whole fresh blood. Lymphocytes were gated based on size and scatter in the forward side scatter plot, excluding CD172a+ cells. NK cells and T cells were determined by selecting respectively CD3- and CD3+ cells. Within the CD3- cells, NK cells were gated as CD56+ dim and bright cells. Within the CD3+ cells CD8+ (Tc cells) and CD4+ (Th cells) and CD8+ CD4+ cells (immature T cells) were selected. Within both CD8+ and CD4+ population, the percentage of CD45RO was measured. Zebra diagrams were then used for setting these gates at the upper limit of the dense negative core.

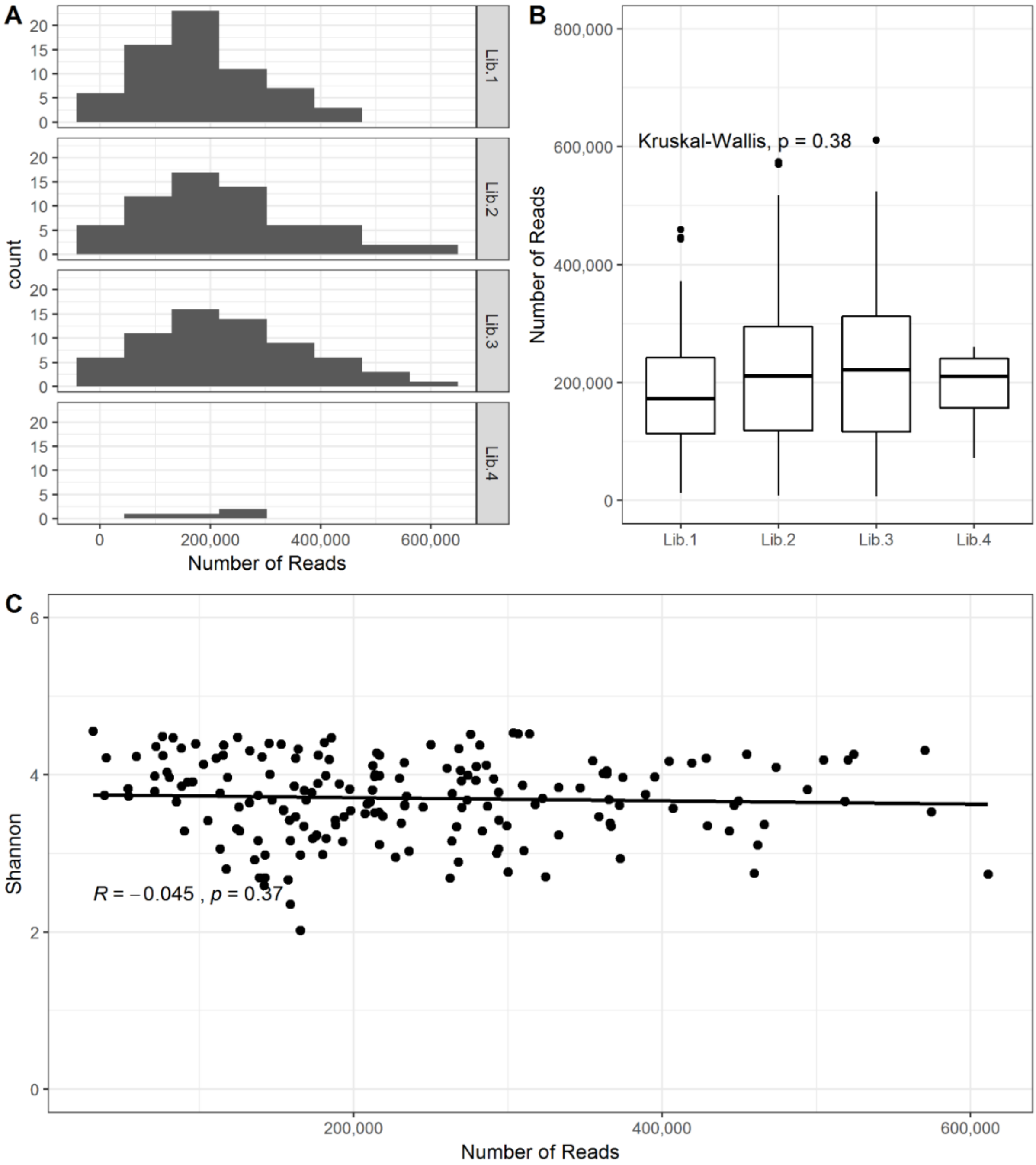

Figure S9. Assessing the effect of the different sequencing batches. A) Histogram with the number of reads obtained per sequencing batch. B) Kruskal-Wallis rank comparison did not reveal differences between the four batches in regard to the obtained number of reads. C) Pearson correlation between the Shannon diversity index and the number of reads for each sample.

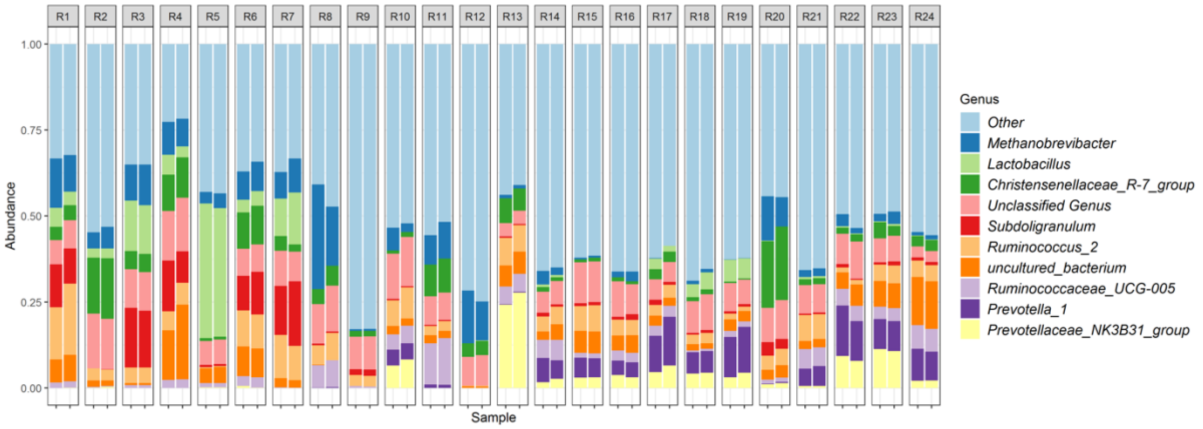

Figure S10. Compositional profiles at genus level for the 24 samples sequenced in duplicates.

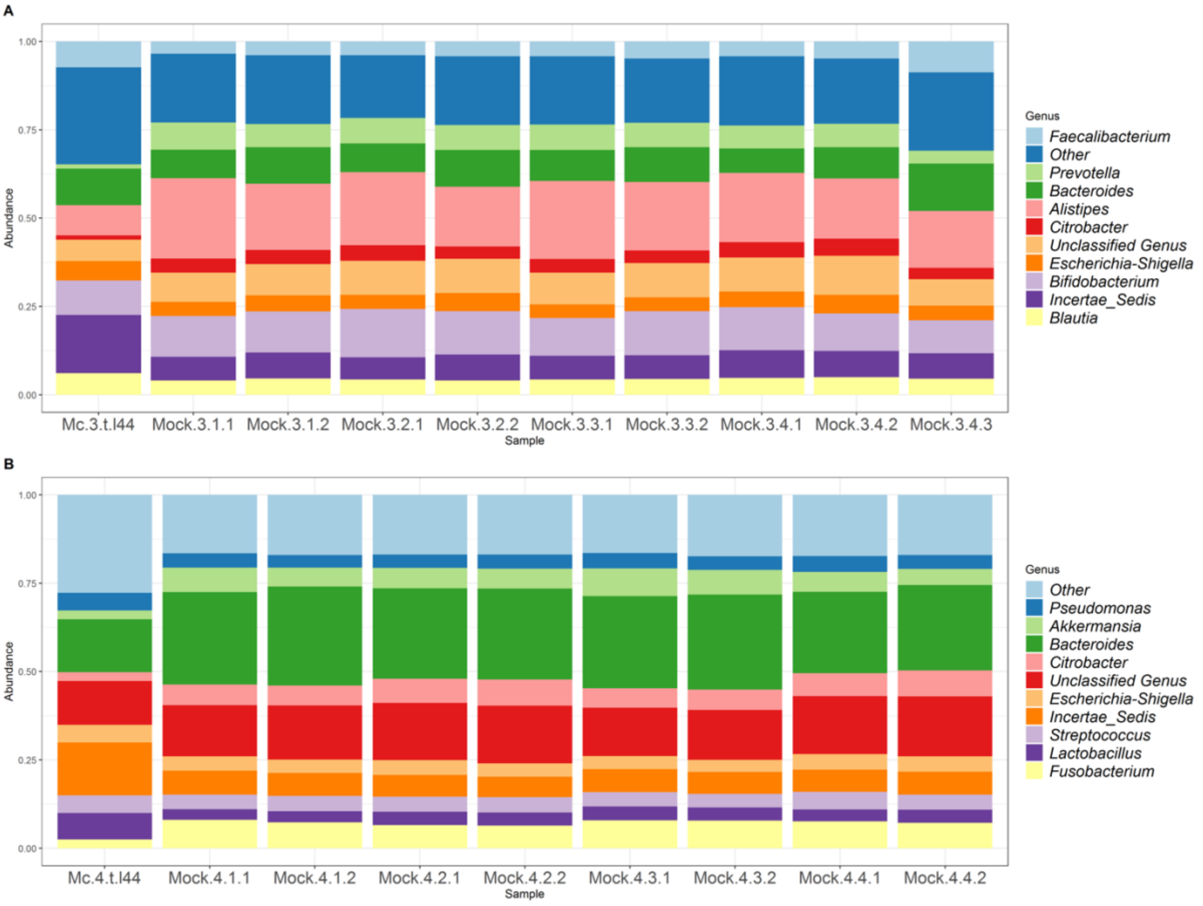

Figure S11. Compositional profiles at genus level for the two mock communities included in our analysis. (A) Mock community 1 (n=9) and (B) mock community 2 (n=8). The theoretical composition for each community is indicated in the first bar graph. Pearson correlation analyses indicated strong correlation between the theoretical composition and the sequenced mocks (R=0.74 for Mock 3 and R=0.84 for Mock 4).

Supplementary Tables

Table S1. PERMANOVA (*p*-values) resulting from the analysis of possible effects of biological (B.Sow) and cross foster sows (CF.Sow) on the development of piglets within each timepoint. Neither biological nor cross foster sows had a significant effect on the microbiota composition of the piglets.

p-values	B.Sow	CF.Sow
Day 10	0.19	0.17
Day 17	0.5	0.09
Day 23	0.13	0.26
Day 30	0.52	0.13
Day 51	0.21	0.22
Day 55	0.32	0.11

Table S2. Composition of the piglet weaner synthetic diet low in fibre.

Ingredient	%
Wheat starch native	50.2
Maize heat treated	10.0
Casein	8.8
Potato protein	4.2
Cellulose	5.0
Dextrose	2.0
Sugar	1.5
Soybean oil	4.2
Amino acids, minerals and vitamins	5.1

Table S3. Nutritional values of the synthetic diet for a swine net energy of 11.0 mega Joules/kg BW.

Nutrition component	%
Protein	18.9 %
Fat	5.1%
Fiber	4.3%
Ash	4.2%
Starch	50.9%
Non Starch Polysaccharides	8.0%
Sodium	0.3%
Calcium	0.6%
Phosphorus	0.5%
Lysine	1.3%
Methionine	0.6%

Table S4. Antibody specifications.

Specificity	Clone name	Fluorchrome	Specificity	Dilution*	Supplier
CD3e	BB23-8E6-8C8	Phycoerytrine (PE)-Cy5	Mouse anti-porcine	5/100	Becton Dickinson (BD), Franklin Lakes, NJ, USA
CD4	74-12-4	PerCP Cy5.5	Mouse anti-porcine	5/100	BD
CD8a	76-2-11	FITC	Mouse anti-porcine	2/100	BD
CD172a	74-22-15A	PE	Mouse anti-porcine	0.1/100	BD
CD45RO	UCHL1	Brilliant Violet (BV) 421	Mouse anti-human, porcine cross-reactive	5/100	BD
CD56	MEM-188 non-biotinylated		Mouse anti-human, porcine cross-reactive	5/100	BioLegend, San Diego, CA, USA
		Streptavidin-BV 785		1/100#	BioLegend

** Dilution used in a total volume of 100µL PBS 10%hiFCS (v/v) supplemented with 10% (v/v) mouse serum*

# without mouse serum

Table S5. Pearson correlation between the duplicate samples with the corresponding Pearson rho for each pair.

Sample Name	Replicate Number	Pearson Coefficients
G1.D23.P1	R1	0.95
G1.D23.P2	R2	0.98
G1.D23.P8	R3	1.00
G1.D23.P6	R4	0.93
G1.D23.P5	R5	1.00
G1.D23.P3	R6	0.98
G1.D23.P7	R7	0.96
G1.D51.P7	R8	0.92
G1.D54.P6	R9	0.99
G1.D54.P5	R10	0.94
G1.D54.P7	R11	0.99
G2.D17.P3	R12	0.99
G2.D54.P5	R13	1.00
G2.D54.P3	R14	0.94
G2.D54.P2	R15	1.00
G2.D54.P4	R16	0.96
G3.D51.P5	R17	0.93
G3.D51.P7	R18	0.94
G3.D51.P6	R19	0.97
G4.D30.P2	R20	0.98
G4.D51.P4	R21	1.00
G4.D51.P2	R22	0.92
G4.D51.P3	R23	0.95
G4.D51.P1	R24	1.00
